# Occupational exposure to gases/fumes and mineral dust affect DNA methylation levels of genes regulating expression

**DOI:** 10.1093/hmg/ddz067

**Published:** 2019-04-02

**Authors:** Diana A van der Plaat, Judith M Vonk, Natalie Terzikhan, Kim de Jong, Maaike de Vries, Sacha La Bastide-van Gemert, Cleo C van Diemen, Lies Lahousse, Guy G Brusselle, Ivana Nedeljkovic, Najaf Amin, BIOS Consortium, Bastiaan T Heijmans, Peter A C ‘t Hoen, Joyce van Meurs, Aaron Isaacs, Rick Jansen, Lude Franke, Dorret I Boomsma, René Pool, Jenny van Dongen, Jouke J Hottenga, Marleen MJ van Greevenbroek, Coen D A Stehouwer, Carla J H van der Kallen, Casper G Schalkwijk, Cisca Wijmenga, Lude Franke, Sasha Zhernakova, Ettje F Tigchelaar, P Eline Slagboom, Marian Beekman, Joris Deelen, Diana van Heemst, Jan H Veldink, Leonard H van den Berg, Cornelia M van Duijn, Bert A Hofman, Aaron Isaacs, André G Uitterlinden, Joyce van Meurs, P Mila Jhamai, Michael Verbiest, H Eka D Suchiman, Marijn Verkerk, Ruud van der Breggen, Jeroen van Rooij, Nico Lakenberg, Hailiang Mei, Maarten van Iterson, Michiel van Galen, Jan Bot, Dasha V Zhernakova, Rick Jansen, Peter van’t Hof, Patrick Deelen, Irene Nooren, Peter A C ‘t Hoen, Bastiaan T Heijmans, Matthijs Moed, Lude Franke, Martijn Vermaat, Dasha V Zhernakova, René Luijk, Marc Jan Bonder, Maarten van Iterson, Patrick Deelen, Freerk van Dijk, Michiel van Galen, Wibowo Arindrarto, Szymon M Kielbasa, Morris A Swertz, Erik W van Zwet, Rick Jansen, Peter-Bram’t Hoen, Bastiaan T Heijmans, Bastiaan T Heijmans, Peter A C ‘t Hoen, Joyce van Meurs, Aaron Isaacs, Rick Jansen, Lude Franke, Dorret I Boomsma, René Pool, Jenny van Dongen, Jouke J Hottenga, Marleen MJ van Greevenbroek, Coen D A Stehouwer, Carla J H van der Kallen, Casper G Schalkwijk, Cisca Wijmenga, Lude Franke, Sasha Zhernakova, Ettje F Tigchelaar, P Eline Slagboom, Marian Beekman, Joris Deelen, Diana van Heemst, Jan H Veldink, Leonard H van den Berg, Cornelia M van Duijn, Bert A Hofman, Aaron Isaacs, André G Uitterlinden, Joyce van Meurs, P Mila Jhamai, Michael Verbiest, H Eka D Suchiman, Marijn Verkerk, Ruud van der Breggen, Jeroen van Rooij, Nico Lakenberg, Hailiang Mei, Maarten van Iterson, Michiel van Galen, Jan Bot, Dasha V Zhernakova, Rick Jansen, Peter van ‘t Hof, Patrick Deelen, Irene Nooren, Peter A C ‘t Hoen, Bastiaan T Heijmans, Matthijs Moed, Lude Franke, Martijn Vermaat, Dasha V Zhernakova, René Luijk, Marc Jan Bonder, Maarten van Iterson, Patrick Deelen, Freerk van Dijk, Michiel van Galen, Wibowo Arindrarto, Szymon M Kielbasa, Morris A Swertz, Erik W van Zwet, Rick Jansen, Peter-Bram ‘t Hoen, Bastiaan T Heijmans, Hans Kromhout, Roel C H Vermeulen, Dirkje S Postma, Cornelia M van Duijn, H Marike Boezen

**Affiliations:** 1 Department of Epidemiology; 2 Groningen Research Institute for Asthma and COPD (GRIAC), University Medical Center Groningen, University of Groningen, Groningen, The Netherlands; 3 Department of Respiratory Medicine, Ghent University Hospital, Ghent, Belgium; 4 Department of Respiratory Medicine, Erasmus Medical Center, Rotterdam, the Netherlands; 5 Department of Genetics, University Medical Center Groningen, University of Groningen, Groningen, The Netherlands; 6 Division Environmental Epidemiology, Institute for Risk Assessment Sciences, Utrecht University, Utrecht, The Netherlands; 7 Department of Pulmonary Diseases, University Medical Center Groningen, University of Groningen, Groningen, The Netherlands

## Abstract

Many workers are daily exposed to occupational agents like gases/fumes, mineral dust or biological dust, which could induce adverse health effects. Epigenetic mechanisms, such as DNA methylation, have been suggested to play a role. We therefore aimed to identify differentially methylated regions (DMRs) upon occupational exposures in never-smokers and investigated if these DMRs associated with gene expression levels. To determine the effects of occupational exposures independent of smoking, 903 never-smokers of the LifeLines cohort study were included. We performed three genome-wide methylation analyses (Illumina 450 K), one per occupational exposure being gases/fumes, mineral dust and biological dust, using robust linear regression adjusted for appropriate confounders. DMRs were identified using comb-p in Python. Results were validated in the Rotterdam Study (233 never-smokers) and methylation-expression associations were assessed using Biobank-based Integrative Omics Study data (*n* = 2802). Of the total 21 significant DMRs, 14 DMRs were associated with gases/fumes and 7 with mineral dust. Three of these DMRs were associated with both exposures (*RPLP1* and *LINC02169* (2×)) and 11 DMRs were located within transcript start sites of gene expression regulating genes. We replicated two DMRs with gases/fumes (*VTRNA2-1* and *GNAS*) and one with mineral dust (*CCDC144NL*). In addition, nine gases/fumes DMRs and six mineral dust DMRs significantly associated with gene expression levels. Our data suggest that occupational exposures may induce differential methylation of gene expression regulating genes and thereby may induce adverse health effects. Given the millions of workers that are exposed daily to occupational exposures, further studies on this epigenetic mechanism and health outcomes are warranted.

## Introduction

Daily, millions of workers worldwide are exposed to chemical agents, fumes and (in)organic dusts (1). The leading occupational causes of death in 2000 were unintentional injuries (41%), chronic obstructive pulmonary disease (COPD, 40%) and lung cancer (13%) (1). This is not remarkable, since the skin and the lungs are most directly exposed to occupational pollutants, which could be prevented by implementing protective measures. Studies focusing on specific occupations, like pig farmers, miners, construction and textile workers, found associations between job-specific exposures and a faster annual decline in lung function (FEV_1_) (2–4). In addition, we have previously shown that exposure to gases/fumes, mineral and biological dust is associated with small and large airways obstruction (5,6).

Even though occupational exposures are common, it is still largely unknown how these exposures are involved in (lung) disease development. Epigenetic mechanisms such as DNA methylation have been suggested to play a role, and researchers have therefore advocated the importance of epigenetic studies into environmental exposures and lung health (7). Environmental exposures, like occupational exposures, induce changes in DNA methylation levels, which can affect gene expression, possibly aiding in disease development (8). DNA methylation is the addition of a methyl group to the DNA without altering its sequence. This usually occurs at sites where a cytosine base is adjacent to a guanine base (CpG) and can have a regulatory function on gene expression (9). Several small studies showed suggestive evidence that specific compounds found in occupational exposures, like cadmium, lead and mercury, affect DNA methylation (8,10–12).

To date, no large hypothesis-free genome-wide DNA methylation studies assessing the association between occupational exposures and DNA methylation levels have been performed. We therefore aimed to identify differentially methylated CpG sites (CpGs) and differentially methylated regions (DMRs) associated with occupational exposure to gases/fumes, mineral dust and biological dust, and to assess the effects of these regions on gene expression levels. To determine the effects of occupational exposures independent of smoking exposure, the analyses were restricted to never-smokers.

## Results

### Population characteristics

Our identification cohort comprised 903 never-smokers of the LifeLines cohort study with genome-wide DNA methylation data (Illumina 450 K) and complete data on all covariates (13). The validation cohort comprised 233 never-smokers of the Rotterdam Study and the characteristics of both cohorts are presented in Table 1, with additional characteristics of LifeLines subjects shown in Supplementary Material, Table S1 (14).

**Table 1:** Characteristics of the never-smokers included in the LifeLines cohort study (discovery cohort) and the Rotterdam Study (validation cohort)

	**LifeLines**	**Rotterdam Study**
*N* with no missing data	903	233
Males, *N* (%)	508 (57)	100 (43)
Age (years), median (min–max)	46 (18–80)	57 (47–89)
Occupational exposure, *N*	**No/low/high**	**No/low/high**
Gases/fumes	637/150/116	177/51/5
Mineral dust	673/105/125	210/20/3
Biological dust	720/69/114	N/A

SD, standard deviation; N/A, not applicable. See Supplementary Material, Table S1 for the characteristics of the LifeLines cohort separately per exposure level. NB: The LifeLines sample is a selected population, not a sample from the general population.

**Figure 1:**
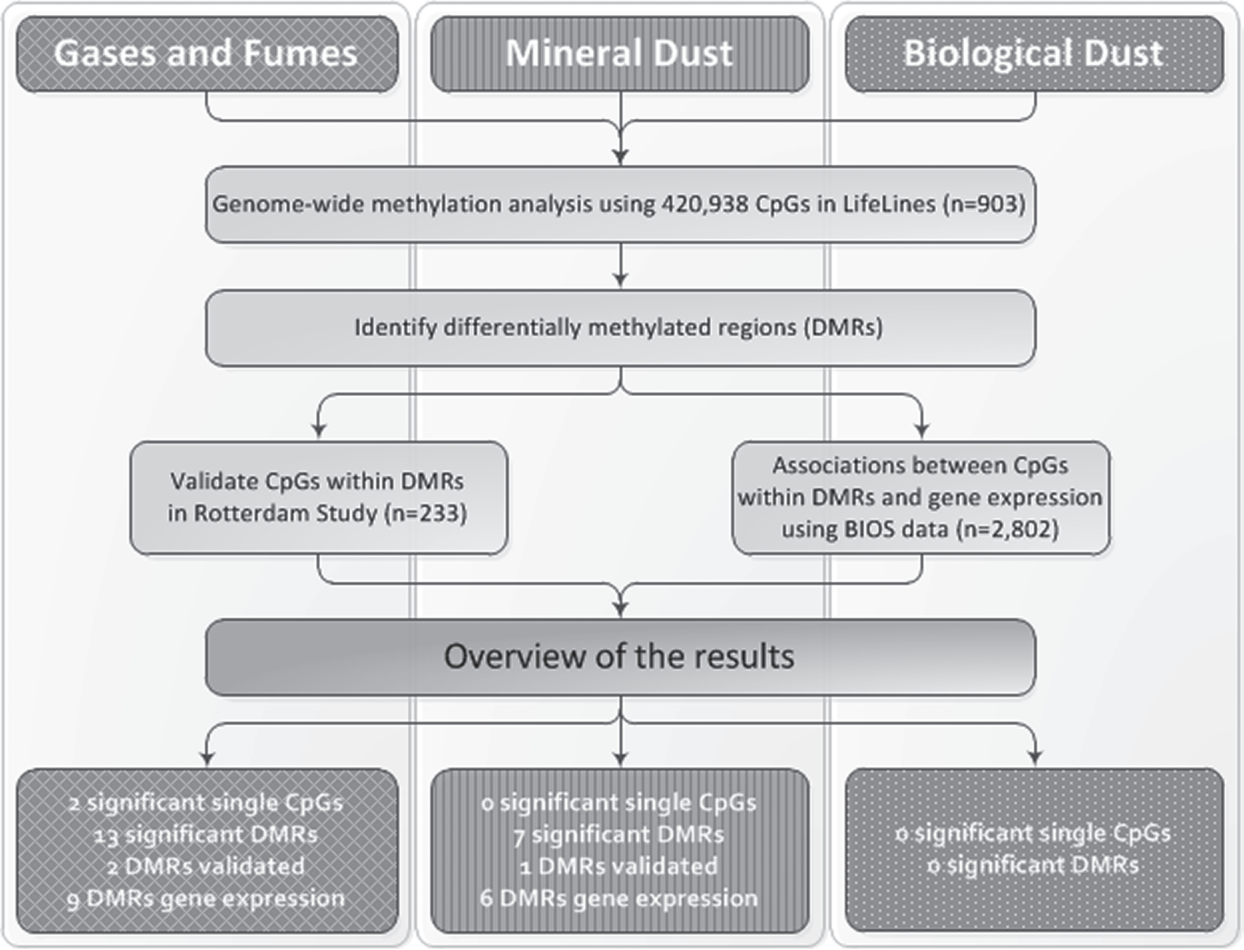
Overview of the performed analyses and results per occupational exposure. All analyses were performed for the three exposures in never-smokers.

Three genome-wide methylation analyses were performed in never-smokers, one per occupational exposure being gases/fumes, mineral dust and biological dust, and consequently DMRs were identified. We present the results of our analyses per occupational exposure, and see Figure 1 for an overview of the performed analyses and corresponding results. The results of all analyses can be found in the supplementary Excel file including all supplementary tables and the Manhattan plots are shown in Supplementary Material, Figure S1.

**Table 2:** Results of the CpGs within the replicated DMRs associated with occupational exposures in never-smokers

				**N**	**Annotation**			***P***		**LifeLines**			**Rotterdam Study**		
**DMR**	**Chr**	**Start**	**End**	**Probes**	**Gene**	**Feature**	**Island**	**Region**	**CpG**	**Beta**	**SE**	***P***	**Beta**	**SE**	***P***
Gases/fumes															
GN4	chr5	135 416 331	135 416 579	7	*VTRNA2–1*	TSS200	Island	2.68^*^10^−5^	cg18678645	−2.84	0.83	5.96^*^10^−4^	−5.68	3.49	0.103
									cg06536614	−1.31	0.56	1.90^*^10^−2^	−3.25	2.47	0.188
									cg26328633	−1.88	0.59	1.50^*^10^−3^	−5.81	2.67	**0.029**
									cg25340688	−1.96	0.63	1.80^*^10^−3^	−2.04	2.89	0.479
									cg26896946	−1.10	0.44	1.18^*^10^−2^	−2.33	2.33	0.318
									cg00124993	−1.59	0.55	3.75^*^10^−3^	−6.23	3.32	0.061
									cg08745965	−2.21	0.84	8.84^*^10^−3^	−6.87	4.50	0.127
GN13	chr20	57 427 713	57 427 880	6	*GNAS*; *GNAS-AS1*	Intron; TSS1500	Island	2.44^*^10^−2^	cg04257105	−1.31	0.52	1.25^*^10^−2^	−2.45	1.69	0.146
									cg20528838	−1.20	0.42	4.24^*^10^−3^	−2.52	1.61	0.119
									cg27661264	−1.39	0.46	2.53^*^10^−3^	−3.15	2.24	0.160
									cg19589727	−1.23	0.45	6.27^*^10^−3^	−.52	1.88	0.783
									cg10302550	−1.06	0.54	4.69^*^10^−2^	−4.54	2.31	**0.049**
									cg17414107	−1.32	0.59	2.54^*^10^−2^	−1.81	2.75	0.510
Mineral dust															
MN7	chr17	20 799 408	20 799 694	6	*CCDC144NL; RP11-344E13.3*	TSS200; 5′UTR	Island	1.13^*^10^−3^	cg08458692	−1.06	0.54	5.03^*^10^−2^	−6.07	2.30	**0.008**
									cg14560110	−3.36	1.36	1.37^*^10^−2^	−9.67	6.06	0.111
									cg08288433	−3.05	0.93	1.05^*^10^−3^	−8.33	7.66	0.277
									cg06809326	−2.81	0.92	2.14^*^10^−3^	−10.25	5.65	0.070
									cg22570042	−3.20	1.01	1.48^*^10^−3^	−14.71	7.64	0.054
									cg21980100	−2.05	0.85	1.62^*^10^−2^	−2.90	3.94	0.461

Chr, Chromosome; CpG, DNA-methylation site; SE, standard error.

### Gases/fumes

#### Genome-wide methylation analysis

In the genome-wide methylation analysis in never-smokers of the identification cohort, two single CpGs were epigenome-wide significantly associated with gases/fumes exposure [false discovery rate (FDR) < 0.05] (Supplementary Material, Table S2). These CpGs are annotated to ribosomal protein L37a (*RPL37A)* and Grid2-interacting protein (*GRID2IP*).

#### Identification of DMRs

Thirteen DMRs were significantly associated with exposure to gases/fumes (Supplementary Material, Table S3). The three most significant DMRs are annotated to long intergenic non-protein coding RNA 2169 (*LINC02169*), ribosomal protein lateral stalk subunit P1 (*RPLP1*) and leptin (*LEP*). The genome-wide significant CpG annotated to *RPL37A* was not located within an identified DMR.

#### Validation of the DMRs

**Figure 2:**
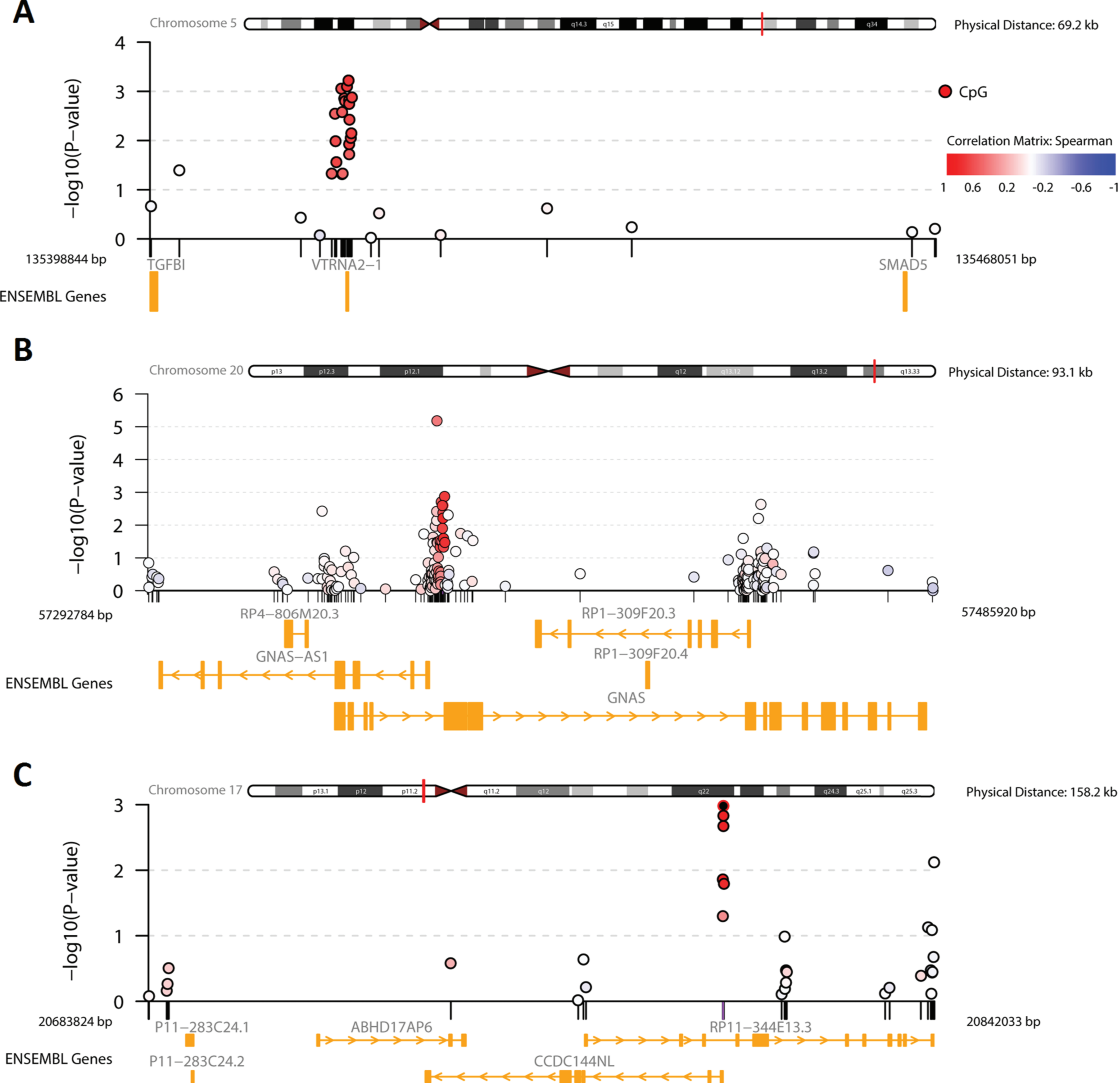
Regional association plots (R package comet) for the three replicated DMRs in never-smokers. **(A)** DMR annotated to *VTRNA2–1*, **(B)** DMR annotated to *GNAS*, and **(C)** DMR annotated to *CCDC144NL*. *x*-axis, megabase (Mb) position on the chromosome; *y*-axis, negative log10 of the *P*-values; dots, CpG sites; and see inset legend for the correlation explanation between CpGs.

In the validation analysis, two DMRs contained a significantly replicated CpG and exposure to gases/fumes was associated with lower methylation levels at these CpGs in both cohorts (Tables 2 and S4). These two DMRs are annotated to Vault RNA 2–1 (*VTRNA2–1*, a.k.a. *MIR886*) and guanine nucleotide-binding protein alpha stimulating activity (*GNAS*) (Fig. 2A and B).

#### Gene expression analysis

We found that CpGs within 9 out of 14 DMRs were significantly associated with differential gene expression, the direction of effect was predominantly negative. Table 3 presents the significant methylation–expression associations of CpGs within replicated DMRs. For the results of all DMRs, see Supplementary Material, Table S5. The replicated DMRs annotated to *GNAS* were associated with lower expression of *NPEPL1*.

### Mineral dust

#### Genome-wide methylation analysis and identification of DMRs

No CpGs were genome-wide significantly associated with mineral dust exposure in our identification cohort (FDR < 0.05), but seven DMRs were (Supplementary Material, Table S3). The three most significant hits are annotated to *RPLP1*, *LINC02169* and major histocompatibility Complex class I E (*HLA-E*), and the first two mentioned DMRs were also associated with exposure to gases/fumes.

#### Validation of the DMRs

The DMR annotated to coiled-coil domain containing 144 family, N-terminal like (*CCDC144NL*) contained a significantly replicated CpG and the association between mineral dust exposure and methylation levels was negative in both cohorts (Table 2, Supplementary Material, Table S4 and Fig. 2C).

#### Gene expression analysis

In total, CpGs within six out of seven DMRs were significantly associated with differential gene expression and the direction of effect was predominantly negative (Supplementary Material, Table S6). The replicated DMR annotated to *CCDC144NL* was associated with lower expression of abhydrolase domain containing 17A pseudogene 6 (*ABHD17AP6*), dehydrogenase/reductase 7B (*DHRS7B*) and galectin 9B (*LGALS9B*) (Table 3).

**Table 3:** Results of replicated DMRs in never-smokers who were associated with gene expression levels for genes located within 1 MB of the CpG (*n* = 2802)

**DMR**	**CpG**	**Annotated gene**	**Ensembl_ID**	**Gene**	***B***	**SE**	***P* adjusted**
*Gases/fumes*							
GN13	cg04257105	*GNAS*	ENSG00000254419	*NPEPL1*	−0.428	0.134	2.19*10^−2^
	cg17414107	*GNAS*	ENSG00000254419	*NPEPL1*	−0.388	0.125	2.81*10^−2^
	cg19589727	*GNAS*	ENSG00000254419	*NPEPL1*	−0.580	0.188	3.03*10^−2^
	cg20528838	*GNAS*	ENSG00000254419	*NPEPL1*	−0.472	0.156	3.68*10^−2^
Mineral dust							
MN7	cg06809326	*CCDC144NL*	ENSG00000226981	*ABHD17AP6*	−1.132	0.251	9.44*10^−5^
			ENSG00000109016	*DHRS7B*	0.129	0.044	2.53*10^−2^
	cg08288433	*CCDC144NL*	ENSG00000226981	*ABHD17AP6*	−0.992	0.231	2.67*10^−4^
	cg14560110	*CCDC144NL*	ENSG00000226981	*ABHD17AP6*	−0.977	0.187	2.57*10^−6^
			ENSG00000170298	*LGALS9B*	0.554	0.168	7.13*10^−3^
	cg21980100	*CCDC144NL*	ENSG00000226981	*ABHD17AP6*	−1.327	0.304	1.92*10^−4^
			ENSG00000109016	*DHRS7B*	0.145	0.053	4.72*10^−2^
	cg22570042	*CCDC144NL*	ENSG00000226981	*ABHD17AP6*	−1.032	0.216	2.57*10^−5^

CpG, DNA-methylation site; *B*, beta; SE, standard error; P adjusted, FDR correct meta-analysis p-value based on genes with available data located within the 1 MB window of the CpG.

### Biological dust

No single CpGs or DMRs were genome-wide significantly associated with biological dust exposure in never-smokers of the identification cohort (FDR < 0.05). Therefore, no validation of results or methylation-expression analyses was performed.

## Discussion

This is the first genome-wide DNA methylation study assessing the association between occupational exposures and DNA methylation. Since it is well known that smoking is associated with extensive changes in DNA methylation levels, we restricted our analyses to never-smokers (15). In these never-smokers, occupational exposure to gases/fumes and to mineral dust was associated with 14 and 7 DMRs, respectively. Three of these DMRs were associated with both gases/fumes and mineral dust (one DMR in *RPLP1* and two DMRs in *LINC02169*). We were able to replicate the result of two DMRs associated with gases/fumes, and one DMR was associated with mineral dust in the Rotterdam Study. These three DMRs were annotated to *VTRNA2-1*, *GNAS* and *CCDC144NL*. CpGs within the DMRs annotated to *GNAS* and *CCDC144NL* were significantly associated with lower expression levels of *NPEPL1* and *ABHD17AP6*, respectively. Moreover, 14 out of 21 DMRs were associated with gene expression levels and 11 DMRs were located within the transcript start sites (TSSs) of a gene. Together, our data suggest that occupational exposures may induce differential DNA methylation at specific genomic locations and this may be a mechanism through which occupational exposures affect health.

Interestingly, the majority of identified DMRs were located within the TSS of a gene; 55.2% and 48.5% of the CpGs within the DMRs associated with gases/fumes and mineral dust, respectively, were located in the TSS, compared to 25.7% of all included CpGs in the study. The three replicated DMRs were also located in the TSS, of which two were also associated with gene expression levels (*GNAS* and *CCDC144NL*). The general idea of the function of DNA methylation at these TSSs is that it blocks the initiation of transcription and thereby lowers gene expression (9). In the current study, we observed that occupational exposure is associated with lower DNA methylation levels which in turn are associated with higher gene expression levels for most DMRs associated with gene expression levels. This observation thus corroborates our knowledge of the function of DNA methylation at TSSs. Moreover, several of the DMRs associated with gene expression were not associated with the annotated gene. This is partly due to the fact that for 11 of our identified DMRs no gene expression data was available for the annotated gene, including the replicated DMR annotated to *VTRNA2-1*. For others, CpGs within a DMR were nominally associated with expression levels of the annotated gene but did not survive the multiple testing correction (e.g. the replicated DMR annotated to *GNAS*).

Another intriguing observation is that several DMRs that we identified are annotated to or associated with the expression of genes with unknown function, RNA genes or pseudogenes, like *CCDC144NL*, *ABHD17AP6*, *NPEPL1*, *RP11-373 N24.2* and *LINC02169*. It is therefore challenging to understand the relation between these genes and occupational exposures. Long non-coding RNAs (lncRNAs) are known to play a role in gene expression regulation during development, cell differentiation, genomic imprinting and sex chromosomal dosage compensation (16). The gene *ZSCAN26* is a zinc finger (transcription factor) and may therefore also be involved in gene expression regulation (17). In addition, multiple microRNAs and lncRNAs were shown to be key regulators of gene expression in lung diseases such as asthma and COPD (18). These might even be biomarkers or therapeutic targets, but more research into the function of these genes is warranted. For your interest, results of Kyoto Encyclopedia of Genes and Genomes (KEGG) pathways and Gene Ontology (GO) term enrichment analyses are included in Supplementary Material, Tables S7 and S8. Overall, gene expression changes could drive DNA methylation changes due to cellular differentiation as a response to occupational exposures. However, it is more likely that occupational exposures may affect regulation of gene expression by changing DNA methylation levels of particular genes that regulate the expression of other genes.

Interestingly, the three DMRs annotated to *RPLP1* and *LINC02169* (2×) were identified in both the gases/fumes and mineral dust analyses. In addition, CpGs annotated to *VTRNA2-1* were also associated with occupational exposure to pesticides in our previous study (13). *RPLP1* is a ribosomal protein regulating translation and *VTRNA2-1* is indirectly also related to the innate immune response, since it was shown to inhibit protein kinase R (*EIF2AK2*) (19,20). This could indicate that different types of occupational exposures affect similar pathways; alternatively it could result from multiple occupational exposures in specific jobs. For example, construction workers can be exposed to mineral dust and gases/fumes at the same time and crop farmers distribute pesticides over their fields using fuelled machines (gases/fumes exposure). Notably, eight subjects of our cohort were highly exposed to all three occupational exposures and the exposures are moderately to strongly correlated (correlation between gases/fumes and mineral dust = 0.85, between gases/fumes and biological dust = 0.66 and between mineral dust and biological dust = 0.56; Supplementary Material, Table S9). Since we used broad categories of occupational exposures, it was not possible to investigate specific exposure molecules. Occupational exposure levels were also estimated based on current or last held job, and duration of exposure was not taken into account. It is likely that some subjects classified as non-exposed have changed from an ‘exposed’ to a ‘non-exposed’ job, because they experienced adverse effects from the exposures. Therefore, we may have underestimated the effect of occupational exposures on DNA methylation. However, in our cohort on average 72% of the subjects currently exposed to gases/fumes, mineral or biological dust had this job for >5 years and thus had been exposed for a substantial time period in the same job.

Another restriction of our study is the use of blood DNA methylation levels. DNA methylation is cell and tissue specific, and the main route of occupational exposure is via inhalation or skin absorption. However, we have validated a number of CpGs associated with cigarette smoke exposure in lung tissue that were originally identified in whole blood (21). Thus using whole blood could be an efficient way to identify differential DNA methylation upon exposures as an accessible proxy for changes in lung tissue. For your interest, the associations between the lung function measurements FEV_1_, FVC, FEV_1_/FVC and FEF_25–75_ and CpGs within DMRs are provided in Table S10. Furthermore, using a job exposure matrix does not allow to assess specific chemical compounds present in occupational exposures, nor the effect of lifetime exposure. Different types of jobs classified into the same exposure category might contain different chemical compounds as well. Therefore, our results reflect the effect of current or recent occupational exposure on DNA methylation.

In conclusion, our data suggest that occupational exposures may induce differential methylation of genes that regulate gene expression and therefore occupational exposures may induce adverse health effects via this methylation. Several of our identified differentially methylated regions upon occupational exposure to gases/fumes and mineral dust were associated with gene expression levels. Some regions were even associated with two types of occupational exposure. Given the millions of workers that are exposed daily to occupational exposures, further studies on this epigenetic mechanism and health outcomes are warranted. For example, since 40% of the occupational cause of death is due to COPD, especially in developing countries without proper precautions, further studies on this epigenetic mechanism could aid in reducing the global burden of COPD (1).

## Materials and Methods

### Population and measurements

From the LifeLines Cohort Study, 1656 unrelated subjects were selected for DNA methylation assessment (13). Subject selection was based on creating relatively equal-sized groups based on age, smoking, occupational exposures and spirometry. In the current study only never-smokers were included in order to determine the effects of occupational exposures independent of smoking exposure. No, low and high occupational exposures to gases/fumes, mineral dust and biological dust were estimated using the ALOHA+ job exposure matrix, based on current or last held job (6,22). See Figure 1 and supplementary methods for an overview and more detailed information on the methods.

### Genome-wide methylation data and analysis

Illumina Infinium Human Methylation 450 K arrays were used to obtain genome-wide DNA methylation data from whole blood. Samples were processed using the Illumina protocol. Quality control (QC) using *Minfi* and normalization using *DASEN* (*watermelon*) were performed in R (23,24). QC steps included the removal of samples with >1% of all probes having a detection *P* > 0.01, and samples with incorrect sex or SNP prediction. We removed single probes with a detection *P* > 0.01, sex chromosome probes, cross-reactive probes (25), probes measuring SNPs and probes where the CpG itself or the single base extension (SBE) site is a SNP. After QC, we had complete data for 420 938 CpG probes in 903 never-smoking subjects.

We performed three genome-wide methylation analyses, one per occupational exposure being gases/fumes, mineral dust and biological dust. We assessed associations between DNA methylation levels (beta-values ranging from 0 to 1) and the three occupational exposures separately using robust linear regression in R [*MASS* package]. Models included low and high exposure dummy-variables (no exposure as reference), and were adjusted for sex, age, technical variances and differential blood counts (eosinophilic, neutrophilic and basophilic granulocytes, lymphocytes and monocytes, all obtained using standard laboratory techniques). Single CpGs with a FDR adjusted *P* < 0.05 for the high-exposure dummy variable were considered genome-wide significant.

### Identification of DMRs

To identify DMRs, *comb-p* in Python was used (14). Comb-p identifies regions of enrichment by combining adjacent *P*-values into FDR adjusted regional *P*-values using auto-correlation and sliding windows. As input we used *P*-values of the high-exposure dummy variable with the following settings: seed = 0.01 and distance = 300. Each CpG within a DMR with a Šidák-corrected *P* < 0.05 was further investigated (26).

### Validation of DMRs

DMRs identified in LifeLines were validated in the baseline assessment of the third Rotterdam Study cohort (RS-III-1, *n* = 722) (27). Blood DNA methylation levels were measured using Illumina 450 K arrays and processed similar to LifeLines as described elsewhere (28). All CpGs within the DMRs were validated in RS-III-1 and the statistical models (robust linear regression) were similar to LifeLines. Single CpGs and CpGs within the DMRs with a nominal validation *P* < 0.05 and same direction of effect in both cohorts were considered significantly replicated.

### Association between CpGs within DMRs and gene expression

To assess whether the CpGs within the DMRs were associated with gene expression levels, we used data from four population-based cohorts within Biobank-based Integrative Omics Studies (BIOS), from the Biobanking and Biomolecular Resources Research Infrastructure for The Netherlands (BBMRI-NL) (29). In total, 2802 subjects were included in the analyses (independent samples of LifeLines, *n* = 727; Rotterdam Study III-2, *n* = 589; Netherlands Twin Registry, *n* = 900; and Leiden Longevity Study, *n* = 586) (30–33). In each cohort, probesets (read counts from RNA sequencing) within 1 Mb around the CpG were assessed and the linear regression was adjusted for sex, smoking, age and technical variances. Effect estimates of the cohorts were meta-analyzed. CpGs with a meta-analysis FDR-corrected *P*-value below 0.05 were considered significant.

## Supplementary Material

Methylation_jobexp_9-12-18_supp1_clean_ddz067

Supplementry_table_HMG_revised_ddz067

## Notes

*Conflict of Interest statement.* L.L. reports personal fees from Boehringer Ingelheim GmbH, non-financial support from Novartis, grants from AstraZeneca, grants and non-financial support from European Respiratory Society and grants and non-financial support from Belgian Respiratory Society, outside the submitted work. DSP reports: The University of Groningen has received money for D.S.P. regarding a grant for research from Astra Zeneca, Chiesi, Genentec, GSK and Roche. Fees for consultancies were given to the University of Groningen by Astra Zeneca, Boehringer Ingelheim, Chiesi, GSK, Takeda and TEVA. All other authors declare they have no actual or potential competing financial interest.
