# Supplementary material for: Occupational exposure to gases/fumes and mineral dust affect DNA methylation levels of genes regulating expression
Source: Hum Mol Genet. 2019 Apr 2;28(15):2477–85. doi: 10.1093/hmg/ddz067 (PMC6643643; doi:10.1093/hmg/ddz067)
Supplement: Methylation_jobexp_9-12-18_supp1_clean_ddz067 [file methylation_jobexp_9-12-18_supp1_clean_ddz067.docx]

# Supplementary material

**Occupational exposure to gases/fumes and mineral dust affect DNA methylation levels of genes regulating expression**

**Authors:** Diana A van der Plaat, PhD^1;2^; Judith M Vonk; PhD^1;2*^; Natalie Terzikhan^3-5*^; Kim de Jong, PhD^1;2^; Maaike de Vries, PhD^1;2^; Sacha La Bastide-van Gemert, PhD^1^; Cleo C van Diemen, PhD^6^; Lies Lahousse, PhD^3-5^; Prof. Guy G Brusselle, MD PhD^3-5^; Ivana Nedeljkovic, MD^5^; Najaf Amin, PhD^5^; BBMRI BIOS†; Prof. Hans Kromhout, PhD^7^; Prof. Roel C H Vermeulen, PhD^7^; Prof. Dirkje S Postma, MD PhD^8;2^; Prof. Cornelia M van Duijn, PhD^5^; Prof. H Marike Boezen, PhD^1;2^

* contributed equally (shared 2^nd^ author)

**Author affiliation:**

^1^University of Groningen, University Medical Center Groningen, Department of Epidemiology, Groningen, the Netherlands.

^2^University of Groningen, University Medical Center Groningen, Groningen Research Institute for Asthma and COPD (GRIAC), Groningen, the Netherlands.

^3^Ghent University Hospital, Department of Respiratory Medicine, Ghent, Belgium.

^4^Erasmus Medical Center, Department of Respiratory Medicine, Rotterdam, the Netherlands.

^5^Erasmus Medical Center, Department of Epidemiology, Rotterdam, the Netherlands.

^6^University of Groningen, University Medical Center Groningen, Department of Genetics, Groningen, the Netherlands.

^7^Utrecht University, Institute for Risk Assessment Sciences, Division Environmental Epidemiology, Utrecht, the Netherlands.

^8^University of Groningen, University Medical Center Groningen, Department of Pulmonary Diseases, Groningen, the Netherlands.

*† Group author: BIOS Consortium (Biobank-based Integrative Omics Study)*

# Supplementary methods

## Population and measurements

LifeLines is a multi-disciplinary prospective population-based cohort study examining in a unique three-generation design the health and health-related behaviours of 167,729 persons living in the North of the Netherlands.(1, 2) It employs a broad range of investigative procedures in assessing the biomedical, socio-demographic, behavioural, physical and psychological factors which contribute to the health and disease of the general population, with a special focus on multi-morbidity and complex genetics. All subjects provided written informed consent and the study was approved by the Medical Ethics Committee of the University Medical Center Groningen, Groningen, the Netherlands. Spirometry was measured using a Welch Allyn Version 1.6.0.489, PC-based SpiroPerfect with Ca Workstation software. From the entire Dutch Lifelines Cohort Study, in total 1,656 subjects were selected for DNA methylation typing. We only selected never- and current-smokers, based on self-reported smoking history of 0 pack-years or greater than 5 pack-years respectively, and excluded ex-smokers to optimize the exposure contrast. In the current study, only never-smokers were included (n=903). A further selection was made to create relatively equal groups based on sex, age, height, occupational exposures, and spirometry. The study sample is therefore not a representation of the LifeLines population. Based on current or last held job, occupational exposure to gases/fumes, mineral dust, and biological dust was estimated using the ALOHA+ Job Exposure Matrix (JEM), as we published previously.(3, 4) First, ISCO-88 job codes were assigned to each subject base on the job description.(5) Second, based on these job codes, the ALOHA+ JEM classified an individual as either likely to be not (0), low (1) or highly (1) exposed. Previous studies have shown that using a JEM is a reliable method to estimate occupational exposures and there is a high correlation with expert opinions and it performs better in estimating job exposures compared to exposure self-reports because it is less likely to be affected by recall bias and differential misclassification.(6–8)

## Genome-wide methylation assay

The 1,656 subjects were randomized over the Illumina Infinium Human Methylation 450K arrays to obtain genome-wide DNA methylation data from whole blood. Details on blood collection and DNA isolation are described elsewhere.(2) A bisulphite conversion was performed using 500ng DNA and the EZ- 96 DNA methylation kit (Zymo research Corporation, Irvine, USA). The Illumina 450K protocol (Illumina Inc., San Diago, USA) was used to further process the samples. Quality-control (QC) steps included the removal of samples with >1% of all probes having a detection p-value >0.01, and samples with an incorrect sex or SNP prediction, using the *Minfi* package in R statistical software.(9) We removed single probes with a detection p-value >0.01, sex chromosome probes, cross-reactive probes, probes measuring SNPs, and probes where the CpG itself or the single base extension (SBE) site is a SNP.(10) Normalization was performed using DASEN implemented in the *wateRmelon* package in R.(11) After QC, we had complete data for 1,561 subjects and 420,938 CpG probes. Of these, only never-smokers were included in the current-study (n=903).

## Genome-wide methylation analysis

We assessed the association between DNA methylation levels, expressed as beta-values ranging from 0 to 1, and the three occupational exposures separately (gases/fumes, mineral dust, and biological dust) using robust linear regression in R (MASS package). The models included dummy-variables for low and high exposure with no exposure as reference, and were adjusted for sex, age, technical variances (7 Principal Components (PCs)), and differential blood counts (5 cell types). Technical variances were estimated by performing a PC-analysis using the control probes and seven PCs were included in the final model (each PC explained >1% of the variance and together the 7 PCs explained 95.5% of the variance (Table E1)).(12) Interestingly, the position on the chip (Sentrix_position) was an important determinant of the measured variance (Figure E1 and E2). The differential blood counts included eosinophilic, neutrophilic, and basophilic granulocytes, lymphocytes and monocytes, and were obtained using standard laboratory techniques. Estimated cell counts using the Houseman method were therefore not used.(13) We assessed the association between the occupational exposures and genome-wide DNA methylation in never-smokers only, in order to determine the effects of occupational exposures independent of smoking exposure. Single CpGs with a false discovery rate (FDR) adjusted p-values <0.05 for the high exposure dummy-variable were considered genome-wide significant.

****Table E1. Principal component analysis performed in the control probes.****

|  | **PC1** | **PC2** | **PC3** | **PC4** | **PC5** | **PC6** | **PC7** |
| --- | --- | --- | --- | --- | --- | --- | --- |
| Proportion Of Variance | 70.8% | 9.9% | 6.2% | 3.7% | 2.2% | 1.5% | 1.1% |
| Cumulative Proportion | 70.8% | 80.7% | 86.9% | 90.6% | 92.8% | 94.3% | 95.5% |

*PC = Principal component*


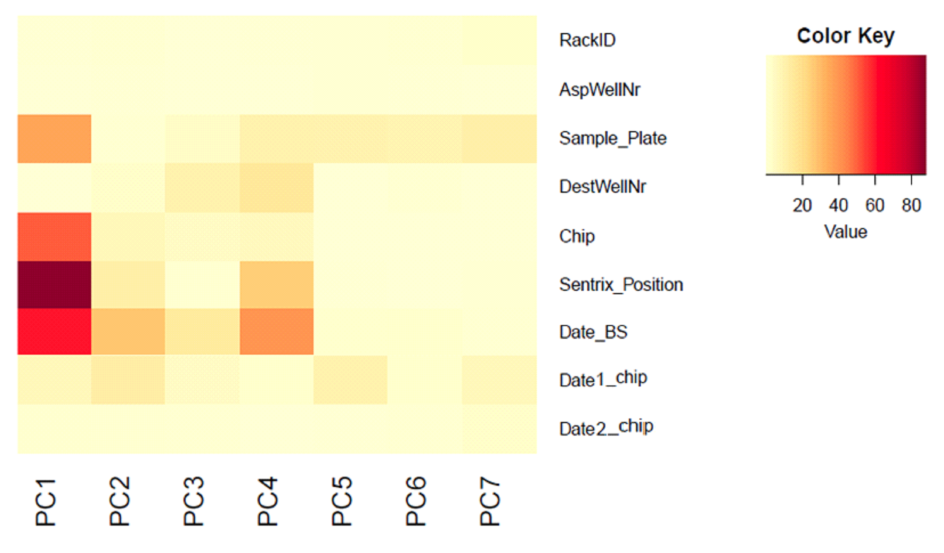


Figure E1. Heatmap of the correlation between the seven principal components and possible known batch effects. The more red the color is, the higher the correlation between variables.


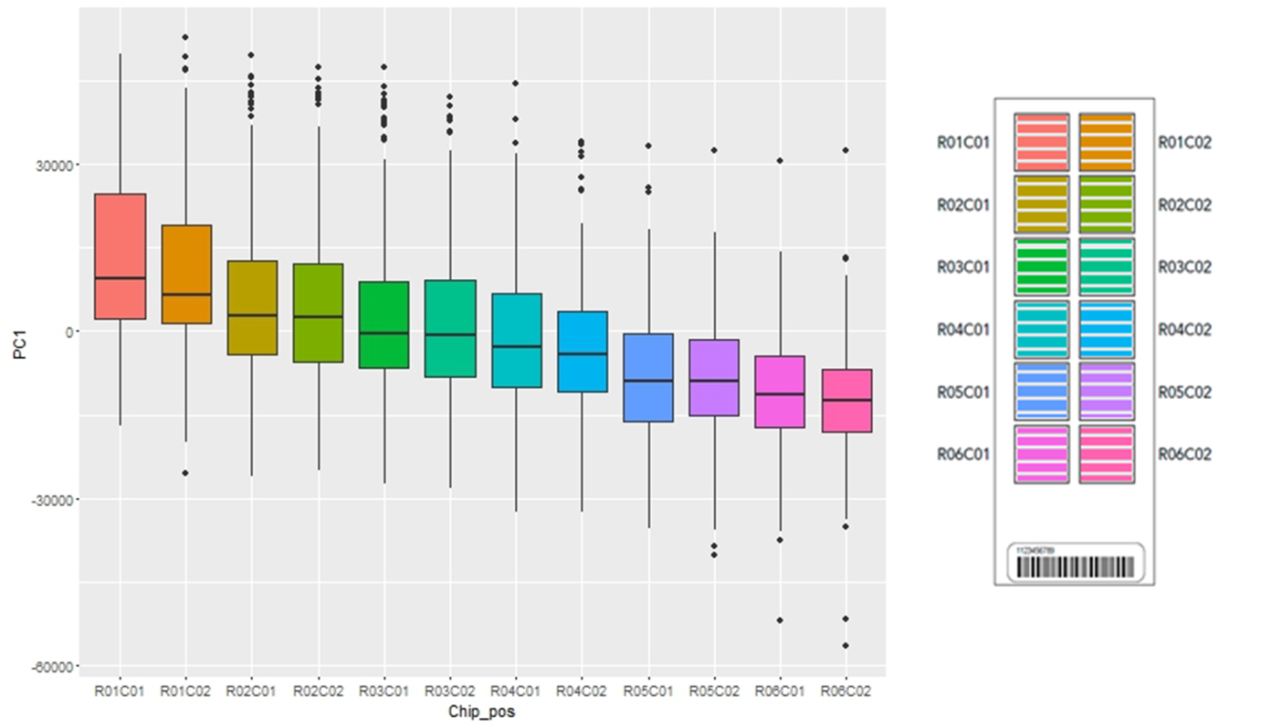


Figure E2. Plot of principal component 1 versus the position on the chip.

## Identification of differentially methylated regions (DMRs)

To identify DMRs, comb-p in python was used.(14) Comb-p finds regions of enrichment by combining adjacent p-values into FDR adjusted regional p-values using auto-correlation and sliding windows. As input we used the p-values of the dummy-variable indicating high exposure and the following settings were used: seed=0.01 and distance=300 (based on the examples provided in the comb-p documentation).(14) The seeds indicates the minimal required p-value to start a region and the distance indicates the number of base pairs around CpG wherein the program will try to find another significant CpG. All DMRs with a Šidák corrected p-value <0.05 were further investigated for validation and gene expression.(15) A DMR could comprise of 1 or more CpGs.

## Validation of DMRs

The results of each CpG located within a DMR, and in addition all CpGs with a false discovery rate (FDR) adjusted p-values <0.05 for the high exposure dummy-variable, were validated in the Rotterdam cohort first visit of the third cohort (RS III-1, 233 never-smokers). The cohort characteristics and design have been described by Hofman et al.(16) Blood DNA methylation levels were measured using Illumina 450K arrays and processed similar to LifeLines as described elsewhere.(17) The used models in RS were similar to the models used in LifeLines: DNA methylation ~ exposure_low + exposure_high + sex + age + monocytes + granulocytes + lymphocytes + array + position. CpGs within a DMR with a nominal validation p-value <0.05 and with the same direction of effect in both cohorts were considered to be significantly replicated.

## Association between CpGs within DMRs and gene expression

To assess whether the CpGs within the DMRs were associated with gene expression levels, we used data of the Biobanking and Biomolecular Resources Research Infrastructure for The Netherlands (BBMRI-NL), BIOS (Biobank-based Integrative Omics Studies) project.(18) GWAS, RNA-sequencing (>15 M paired end reads) and genome-wide DNA methylation (Illumina 450k arrays) data are available within this biobank. Details on how this data was generated can be found on the website and cohort specific publications (<http://bios-vm.bbmrirp3-lumc.surf-hosted.nl/BBMRIomics/index.html>).(19–22) Access to BIOS data is available for all researchers by application to the BIOS Data Access Committee ([www.bbmri.nl/acquisition-use-analyze/bios/](http://www.bbmri.nl/acquisition-use-analyze/bios/)).

Data were used from four population based cohorts within BIOS, Netherlands Twin Registry (n=900), Leiden Longevity Study (n=586), and independent samples of LifeLines (n=727) and the Rotterdam Study III-2 (n=589).(19–22) In each cohort genes with available data (read counts from RNA sequencing) within 1Mb around the CpG were assessed and the linear regression model was adjusted for sex, smoking, age and batch effects (cohort specific PCs). The results of the cohorts were meta-analysed using a random-effect model based on the effect estimates and weighted by the standard errors. CpGs with a meta-analysis p-value below the Bonferroni corrected threshold (p=0.05/number of genes with available data in 1MB window) were considered significant.

## References

1. Stolk,R.P., Rosmalen,J.G., Postma,D.S., de Boer,R.A., Navis,G., Slaets,J.P., Ormel,J. and Wolffenbuttel,B.H. (2008) Universal risk factors for multifactorial diseases: LifeLines: a three-generation population-based study. Eur. J. Epidemiol., 23, 67–74.
2. Scholtens,S., Smidt,N., Swertz,M.A., Bakker,S.J., Dotinga,A., Vonk,J.M., van Dijk,F., van Zon,S.K., Wijmenga,C., Wolffenbuttel,B.H., et al. (2015) Cohort Profile: LifeLines, a three-generation cohort study and biobank. Int. J. Epidemiol., 44, 1172–1180.
3. Matheson,M.C., Benke,G., Raven,J., Sim,M.R., Kromhout,H., Vermeulen,R., Johns,D.P., Walters,E.H. and Abramson,M.J. (2005) Biological dust exposure in the workplace is a risk factor for chronic obstructive pulmonary disease. Thorax, 60, 645–651.
4. de Jong,K., Boezen,H.M., Kromhout,H., Vermeulen,R., Postma,D.S., Vonk,J.M. and Study,L.C. (2014) Pesticides and other occupational exposures are associated with airway obstruction: the LifeLines cohort study. Occup. Environ. Med., 71, 88–96.
5. Organization IL. The revised international standard classification of occupations (ISCO-88).
6. Benke,G., Sim,M., Fritschi,L., Aldred,G., Forbes,A. and Kauppinen,T. (2001) Comparison of occupational exposure using three different methods: hygiene panel, job exposure matrix (JEM), and self reports. Appl. Occup. Environ. Hyg., 16, 84–91.
7. Kromhout,H. and Vermeulen,R. (2001) Application of job-exposure matrices in studies of the general population-some clues to their performance. Eur. Respir. Rev., 11, 80–90.
8. Delclos,G.L., Gimeno,D., Arif,A.A., Benavides,F.G. and Zock,J.P. (2009) Occupational exposures and asthma in health-care workers: comparison of self-reports with a workplace-specific job exposure matrix. Am. J. Epidemiol., 169, 581–587.
9. Aryee,M.J., Jaffe,A.E., Corrada-Bravo,H., Ladd-Acosta,C., Feinberg,A.P., Hansen,K.D. and Irizarry,R.A. (2014) Minfi: a flexible and comprehensive Bioconductor package for the analysis of Infinium DNA methylation microarrays. Bioinformatics, 30, 1363–1369.
10. Chen,Y.A., Lemire,M., Choufani,S., Butcher,D.T., Grafodatskaya,D., Zanke,B.W., Gallinger,S., Hudson,T.J. and Weksberg,R. (2013) Discovery of cross-reactive probes and polymorphic CpGs in the Illumina Infinium HumanMethylation450 microarray. Epigenetics, 8, 203–209.
11. Pidsley,R., Y Wong,C.C., Volta,M., Lunnon,K., Mill,J. and Schalkwyk,L.C. (2013) A data-driven approach to preprocessing Illumina 450K methylation array data. BMC Genomics, 14, 293.
12. Lehne,B., Drong,A.W., Loh,M., Zhang,W., Scott,W.R., Tan,S.T., Afzal,U., Scott,J., Jarvelin,M.R., Elliott,P., et al. (2015) A coherent approach for analysis of the Illumina HumanMethylation450 BeadChip improves data quality and performance in epigenome-wide association studies. Genome Biol., 16, 37–015–0600–x.
13. Houseman,E.A., Accomando,W.P., Koestler,D.C., Christensen,B.C., Marsit,C.J., Nelson,H.H., Wiencke,J.K. and Kelsey,K.T. (2012) DNA methylation arrays as surrogate measures of cell mixture distribution. BMC Bioinformatics, 13, 86.
14. Pedersen,B.S., Schwartz,D.A., Yang,I. V and Kechris,K.J. (2012) Comb-p: software for combining, analyzing, grouping and correcting spatially correlated P-values. Bioinformatics, 28, 2986–2988.
15. Sidak,Z. (1967) Rectangular Confidence Regions for the Means of Multivariate Normal Distributions. J. Am. Stat. Assoc., 62, 626–633.
16. Hofman,A., Brusselle,G.G., Darwish Murad,S., van Duijn,C.M., Franco,O.H., Goedegebure,A., Ikram,M.A., Klaver,C.C., Nijsten,T.E., Peeters,R.P., et al. (2015) The Rotterdam Study: 2016 objectives and design update. Eur. J. Epidemiol., 30, 661–708.
17. Ligthart,S., Steenaard,R.V. V, Peters,M.J.J., van Meurs,J.B.B., Sijbrands,E.J.J., Uitterlinden,A.G.G., Bonder,M.J.J., consortium,B., Hofman,A., Franco,O.H.H., et al. (2016) Tobacco smoking is associated with DNA methylation of diabetes susceptibility genes. Diabetologia, 59, 998–1006.
18. Biobanking and BioMolecular Resources research Infrastructure (BBMRI-NL): Biobank-based Integrative Omics Studies (BIOS). 2017; at http://www.bbmri.nl/acquisition-use-analyze/bios/
19. Tigchelaar,E.F., Zhernakova,A., Dekens,J.A., Hermes,G., Baranska,A., Mujagic,Z., Swertz,M.A., Munoz,A.M., Deelen,P., Cenit,M.C., et al. (2015) Cohort profile: LifeLines DEEP, a prospective, general population cohort study in the northern Netherlands: study design and baseline characteristics. BMJ Open, 5, e006772-2014-006772.
20. Hofman,A., van Duijn,C.M., Franco,O.H., Ikram,M.A., Janssen,H.L., Klaver,C.C., Kuipers,E.J., Nijsten,T.E., Stricker,B.H., Tiemeier,H., et al. (2011) The Rotterdam Study: 2012 objectives and design update. Eur. J. Epidemiol., 26, 657–686.
21. Willemsen,G., Vink,J.M., Abdellaoui,A., den Braber,A., van Beek,J.H., Draisma,H.H.H., van Dongen,J., van ’t Ent,D., Geels,L.M., van Lien,R., et al. (2013) The Adult Netherlands Twin Register: twenty-five years of survey and biological data collection. Twin Res. Hum. Genet., 16, 271–281.
22. Westendorp,R.G., van Heemst,D., Rozing,M.P., Frolich,M., Mooijaart,S.P., Blauw,G.J., Beekman,M., Heijmans,B.T., de Craen,A.J., Slagboom,P.E., et al. (2009) Nonagenarian siblings and their offspring display lower risk of mortality and morbidity than sporadic nonagenarians: The Leiden Longevity Study. J. Am. Geriatr. Soc., 57, 1634–1637.


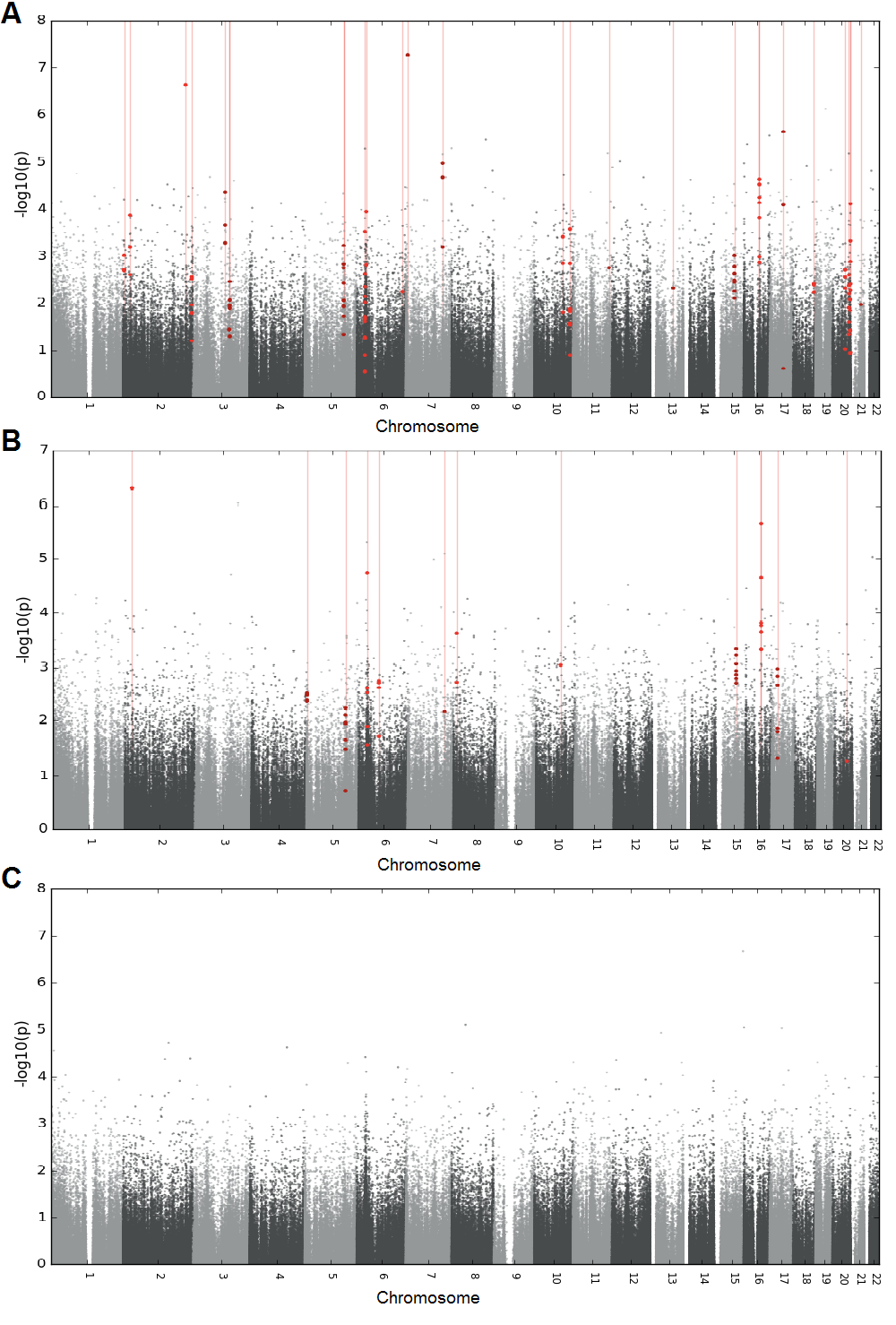
Figure S1: Manhattan plots showing the chromosomal position of the identified DMRs per exposure (red lines). A) Gases and Fumes, B) Mineral dust and C) Biological dust.
